# Diversity of *Listeria monocytogenes* Strains Isolated from Food Products in the Central European Part of Russia in 2000–2005 and 2019–2020

**DOI:** 10.3390/foods10112790

**Published:** 2021-11-12

**Authors:** Ekaterina K. Psareva, Elena A. Liskova, Irina V. Razheva, Yulia K. Yushina, Maria A. Grudistova, Nadezda A. Gladkova, Eugene A. Potemkin, Pavel A. Zhurilov, Elena V. Sokolova, Pavel A. Andriyanov, Olga L. Voronina, Denis V. Kolbasov, Svetlana A. Ermolaeva

**Affiliations:** 1 Federal Research Center for Virology and Microbiology, Branch in Nizhny Novgorod, 603950 Nizhny Novgorod, Russia; liskovaea@mail.ru (E.A.L.); razheva64@bk.ru (I.V.R.); nivigladkova@yandex.ru (N.A.G.); jeka89290462295@gmail.com (E.A.P.); Zhurilov95@bk.ru (P.A.Z.); sokol.e1ena@yandex.ru (E.V.S.); andriyanovpvl@gmail.com (P.A.A.); 2 V.M. Gorbatov Research Center for Food Systems of Russian Academy of Sciences, 109316 Moscow, Russia; yu.yushina@fncps.ru (Y.K.Y.); m.grudistova@fncps.ru (M.A.G.); 3 N.F. Gamaleya National Research Center for Epidemilogy and Microbiology of Ministry of Health of Russia, 123098 Moscow, Russia; olv550@gmail.com; 4 Federal Research Center for Virology and Microbiology, 601125 Volginsky, Russia; kolbasovdenis@gmail.com

**Keywords:** *Listeria monocytogenes*, listeriosis, foodborne pathogen, multilocus sequence typing, invasion

## Abstract

Totally, 45 *L. monocytogenes* strains isolated from meat, poultry, dairy, and fish products in the Central European part of Russia in 2001–2005 and 2019–2020 were typed using a combined MLST and internalin profile (IP) scheme. Strains belonged to 14 clonal complexes (CCs) of the phylogenetic lineages I and II. Almost half of the strains (20 of 45) belonged to six CCs previously recognized as epidemic clones (ECs). ECI and ECV strains were isolated during both studied periods, and ECII, ECIV, ECVI, and ECVII strains were isolated in 2001–2005, but not in 2019–2020. ECI, ECIV, ECV, and ECVII strains were isolated from products of animal origin. ECII and ECVI were isolated from fish. Testing of invasion efficiencies of 10 strains isolated in different years and from different sources and belonging to distinct CCs revealed a statistically significant difference between phylogenetic lineage I and II strains but not between ECs and non-EC CCs or strains differing by year and source of isolation. Strains isolated in 2001–2005 were characterized by higher phylogenetic diversity and greater presentation of ECs and CCs non-typical for natural and anthropogenic environments of the European part of Russia comparatively to isolates obtained in 2019–2020.Closing of the Russian market in 2019–2020 for imported food might be responsible for these differences.

## 1. Introduction

The Gram-positive bacteria *Listeria monocytogenes* causes a severe foodborne disease, listeriosis. Although relatively rare, listeriosis has harsh clinical manifestations and high mortality among groups of risk that include elderly and/or immunocompromised persons, pregnant women, and newborns [1,2]. The most serious manifestations of listeriosis are disorders of the central nervous system and miscarriagesin pregnant women [3]. There are reports of listeriosis outbreaks, both among patients and after eating of listeria infected food products [4,5]. *L. monocytogenes* entering the intestine with contaminated food is able to cross the intestinal epithelial barrier and to colonize lamina propria, from where it spreads to internal organs, primarily to the liver and spleen. If the infection is not controlled at this stage, secondary bacteremia develops and bacteria can cross thebrain–blood or maternal–fetal barrier, infecting the brain orfetus [6]. There are two major virulence factors responsible for intestine and maternofetal barrier crossing: internalin A (InlA) and internaline B (InlB), both belong to the internalin protein family [7]. The genes encoding these proteins are commonly referred to as *inlA* and *inlB*.

Contaminated food products that represent the main *L. monocytogenes* transmission route to humans include meat, poultry, and dairy products, and vegetables [8,9,10]. Sick and carrier animals, farm and food plant environment aresources of food product contamination as *L. monocytogenes* are ubiquitous and highly versatile bacteria that are widely spread in the nature and effectively occupy different ecological niches [11,12,13,14]. Comparative analysis of strains isolated from patients, food products, domestic animals, and environment provides a basis for understanding of *L. monocytogenes* circulation at a particular territory and worldwide [15,16,17,18].

The multilocus sequence typing (MLST) is widely used for *L. monocytogenes* studies of intraspecific diversity and clonal relationships.The MLST scheme is based on sequences of slowly evolving housekeeping genes, characterized by low mutation rates [19,20]. Strains carrying identical sequences of all seven genes are considered as belonging to the same sequence type (ST), and strains differing by only one marker are considered as belonging to one clonal complex (CC) [20]. To further develop a multilocustyping method, a number of multivirulence locus sequence typing (MvLST) schemes were developed that included sequences of virulence genes as additional markers [14,21,22]. Particularly, a combination of sequences of four genes encoding proteins of the internalin family (the IP profile) was demonstrated to further differentiate strains belonging to the same CCs [12,14,22,23].

Large-scale strains analysis proved that the *L. monocytogenes* species has evolved into four phylogenetic lineages that differ in their virulence potential [8,18,24]. Listeriosis in humans and domestic animals is mainly associated with lineage I and II strains while lineage III and IV strains are rare among sources other than the environment. The majority of outbreaks are linked with a few clonal complexes known as epidemic clones (ECs) representing bacteria belonging to a particular clonal complex and most likely of the same originand belonging to the phylogenetic lineage I (EC1, ECII, ECIV, and ECVI) and thelineage II (ECIII, ECV and ECVII) [19,20].

Although epidemic clones are spread worldwide, the prevalence of certain clonal groups have a geographic specific character that may change over time [12,25,26]. Changes in prevalence of certain clonal groups in food products were suggested to be responsible for changes in human listeriosis causative agents, and human activities such as worldwide trading, exchange of agricultural goods, raw food industry materials, and finished products can change prevalence of *L. monocytogenes* clones circulating at a particular geographic region [25,27,28].To further investigate this topic, we compared *L. monocytogenes* strains isolated from food products in Russia during two time periods, 2001–2005 and 2019–2020 when the Russian food market was partly closed due to political and pandemic reasons.

## 2. Materials and Methods

### 2.1. Researched Sources from Meat Products in 2019

In 2019, the *L. monocytogenes* strains were found in food from meat in the Laboratory Center for food and feed testing in the V.M. Gorbatov Research Center for Food Systems of Russian Academy of Sciences. In Russia, methods of food control for the detection of *L. monocytogenes* are described in GOST 32031-2012 “Food products. Methods for the detection of bacteria *Listeria monocytogenes*”. Raw semi-finished poultry meat products were investigated: natural, chopped, and using spices; semi-finished pork and beef products: large, small, and chopped; semi-finished meat products of mixed raw materials (beef and pork): chopped and dough and ready-to-eat meat products. The selection of samples from meat and poultry was carried out in accordance with GOST R ISO 6887-2-2013 “Microbiology of food products and animal feed. Preparation of samples, initial suspension and tenfold dilutions for microbiological studies. Part 2. Special rules for the preparation of meat and meat products ‘and GOST R 54354-2011’ Meat and meat products. General requirements and methods of microbiological analysis”.

A total of 884 samples of semi-finished meat products were analyzed, 106 samples (12%) of which were positive for the bacteria *Listeria monocytogenes*. The highest detectability of *L. monocytogenes* falls on products from poultry meat, averaging 23.3% (52 out of 223 samples studied), in second place in terms of contamination, products from beef meat—19.4% (28 out of 144 samples studied). The share of meat products from mixed raw materials (beef and pork) accounted for 4.8% (16 out of 336 studied samples) of the samples studied, in semi-finished products from pork, *L. monocytogenes* bacteria were found in only 5.5% (10 out of 181 samples studied) of cases.

### 2.2. Culturing Conditions of Bacterial Strains

**Figure 1:**
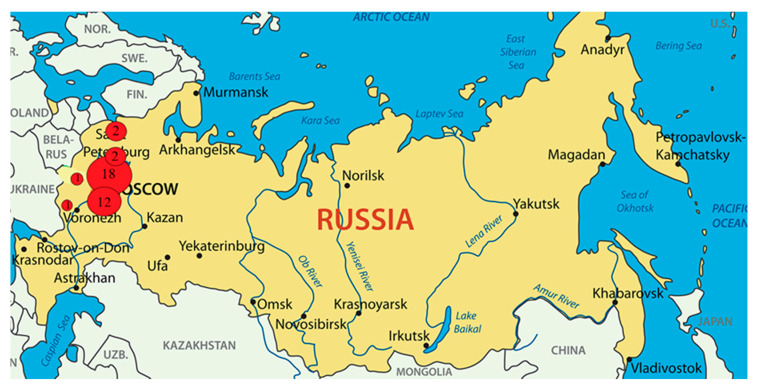
The map shows the strain’s isolation locations.

A total of 45 *L. monocytogenes* strains were investigated from raw meat and poultry (*n* = 22), dairy (*n* = 15), and fish (*n* = 8) products in the territory of the Central European part of Russia (Figure 1) during two periods of 2001–2005 and 2019–2020 (Table S1). Strains of 2001–2005 years were kept in the National Collection of the Federal Research Center for Virology and Microbiology (Volginsky, Russia) and N.F. Gamaleya National Research Center for Epidemilogy and Microbiology of Ministry of Health of Russia (Moscow, Russia). Strains of 2019–2020 years were kept in the collection of V.M. Gorbatov Research Center for Food Systems of Russian Academy of Sciences and N.F. Gamaleya National Research Center for Epidemilogy and Microbiology of Ministry of Health of Russia (Moscow, Russia).

The *L. monocytogenes* strains were cultured on tryptone soya yeast extract agar (TSA-YE, HiMedia, Mumbai, India). Plates were incubated at 37 °C for 24 h; then, colonies were replated for further identification and characterization. All strains were confirmed as *L. monocytogenes* using Microgen Listeria ID (Microgen Bioproducts Ltd., Kimberley, UK). Additionally, colonies from TSA-YE were tested for Gram staining, oxidase tests, catalase reactions, and motility at 20–25 °C (Listeria Motility Medium, HiMedia, Mumbai, India). Genomic DNA of overnight *L. monocytogenes* cultures was isolated using the kit for isolation of genomic DNA from bacterial cells (diaGene, Moscow, Russia).

To prepare bacteria for invasion efficiency testing, the overnight culture of *L. monocytogenes* strains was diluted with tryptone soya yeast extract broth (TSB-YE, HiMedia, Mumbai, India) 1:100 and cultured to an OD = 1.5–1.8 (the phase of exponential growth), then cells were sedimented by centrifugation at 5000 rpm, the cell pellet was washed with sterile phosphate-buffered saline (PBS, Sigma, Saint Louis, MO, USA). Cells were sedimented with centrifugation again, the supernatant was removed, and the cell pellet was resuspended in 10% glycerol in PBS. Bacterial suspension was aliquoted into microtubes, 100 μL each, and stored at −70 °C. Bacterial concentrations were determined the next day after freezing by plating decimal dilutions of the thawed suspension on TSA-YE agar.

### 2.3. Antibiotic Resistance Assays

*L. monocytogenes* strains were tested for resistance to first-line antibiotics using the disc diffusion method on the standard Mueller-Hinton agar (HiMedia, Mumbai, India) containing 5% of defibrinated (mechanically) horse blood and 20 mg/L β-NAD (MH-F). Suspension (or inoculum) was prepared consistent with the EUCAST 2021 manual: colonies from an overnight culture of *L. monocytogenes* were collected with a sterile loop and suspended in saline solution till 0.5 McFarland density was achieved. Inoculated MH-F plates with antibiotics disks were incubated in atmosphere with 5% CO_2_ at 35 ± 1 °C, 18 ± 2 h.The results were taken into account by measuring the diameters of the bacterial suppression zones’ growth with a ruler template. The obtained results were analyzed by the standards of the European Committee for Antimicrobial Susceptibility Testing (EUCAST, 2021), on the basis of which a conclusion was made about the degree of susceptibility of each strain to a specific antibacterial drug and marked in the table as a letter designation: S (susceptible) and R (resistant). Gentamicin (10 µg/disc), Ampicillin (2 µg/disc), and Penicillin G (1 unit/disc) were used (NICF, LLC; St. Petersburg, Russia). In the case of absence breakpoints for certain organisms or agents, EUCAST suggests using interpretation afforded similar species. Since EUCAST 2021 has no breakpoints for the *L. monocytogenes* assessment of susceptibility to gentamicin, we used gentamicin breakpoints for *Staphylococcus aureus*. These antimicrobials are the first-line drugs used for listeriosis treatment as well as one of the most preferable drugs in veterinary medicine in Russia. QC procedures were performed in agreement with the EUCAST 2021 manual. As a strain for QC of MH-F medium and ampicillin (2 µg/disc), penicillin G (1 unit/disc) antimicrobials we used *S. pneumoniae* ATCC 49619 strain (Microbiologics, St. Cloud, MN, USA). For gentamicin disks QC we used *S. aureus* ATCC 29213 (Microbiologics, St. Cloud, MN, USA), because Listeria breakpoints do not cover this agent.

### 2.4. L. monocytogenes Virulence in HEp-2 Cells

We used HEp2 cells (adenocarcinoma cells human larynx) that are widely used to evaluate pathogen invasion efficiency. The HEp2 cells were obtained from Svetlana A. Ermolaeva in the N.F. Gamaleya National Research Center for Epidemilogy and Microbiology of Ministry of Health of Russia (Moscow, Russia). Cells were cultured in Dulbecco’s Modified Eagle Medium (DMEM) (Pan-Eco, Moscow, Russia) supplemented with 10% fetal bovine serum (Thermo Fisher Scientific Inc., Waltham, MA, USA) at 37 °C in 5% CO_2_ atmosphere. To separate cells from the plastic, a dispersing solution of 0.25% trypsin (Pan-Eco, Moscow, Russia) and 0.02% versene (Pan-Eco, Moscow, Russia) in the proportion of 1:3 was used. To prepare a 24-well plate, 1 × 10^5^ cells were added to each well and grown up to a 70–80% monolayer. The number of cells in one well was counted. Bacteria were diluted in serum-free DMEM using a multiplicity of infection from 1:100 to 1:200. The cells were incubated with the *L. monocytogenes* strain for 1 h at 37 °C in an atmosphere with 5% CO_2_, after which they were washed three times with PBS and DMEM medium supplemented with gentamicin 100 μg/mL was added to wells. After 1 h incubation the wells were washed three times with PBS and 200 μL of 1% Triton X-100 were added. Cells were lysed for 5min and serial dilutions were prepared with obtained cell lysates. The dilution was plated on the TSA-YE agar. Colonies were counted after 18 h growth at 37 °C. The efficiency of invasion was calculated as the ratio of intracellular bacteria to the number of bacteria taken for infection.

### 2.5. Multiplex PCR Assay to Serotype L. monocytogenes

We applied a scheme suggested by Doumith et al. to determine the four major *L. monocytogenes* serovars by multiplex PCR assay [29]. The marker genes selected for the multiplex PCR assay were *lmo0737* and *lmo1118*, identified in the sequenced *L. monocytogenes* serovar 1/2a EGDe strain, and *ORF2819* and *ORF2110*, identified in the partial sequence of *L. monocytogenes* 4b strain CLIP 80459. The *prs* gene, specific for strains of the genus Listeria, was targeted for an internal amplification control.

### 2.6. PCR

The MLST scheme for *Listeria monocytogenes* typing is based on sequences of seven housekeeping genes. The original scheme was developed by BIGSdb-*Lm* https://bigsdb.pasteur.fr/listeria/listeria.html (accessed on 10 September 2021). The Encyclo Plus PCR kit (Evrogen, Moscow, Russia) and PCR primers (Evrogen, Moscow, Russia) were used for DNA amplification. PCR products were estimated by 1% agarose gel electrophoresis. Target DNA fragments were purified using Cleanup Standard (Evrogen, Russia). PCR amplification of four internalin genes locuses (*inlA*, *inlB*, *inlC*, and *inlE*) was carried out as previously described [12,14].

### 2.7. Sequencing of PCR Products

Sequencing of both strands of purified PCR products was performed according to the BigDye Terminator 3.1 Cycle Sequencing protocol for the Genetic Analyzer 3130 (Applied Biosystems, Waltham, MA, USA). Electrophoretic separation of DNA was carried out in 50cm capillaries with polymer of POP7 type.

### 2.8. Sequence Analysis

All sequences were checked and assembled in Chromas Lite MFC Application version 2.1.1.0. DNA alignment was conducted in the ClustalX2 software. Dendrograms were constructed with Mega X version 10.1 (https://www.megasoftware.net/, accessed on 21 September 2021). Determination of allelic numbers and profiles (genotypes, sequence types (STs)) was performed through the *L. monocytogenes* MLST database BIGSdb-*Lm* https://bigsdb.pasteur.fr/listeria/listeria.html (accessed on 10 September 2021).

### 2.9. Calculation of Diversity Indexes

The Shannon’s Index (H) was calculated as

H = −Σ((n_i_/N) × log_2_ × (n_i_/N)),

where n_i_ is the number of strains belonging to the CC_i_, and N is the total number of strains belonging to all CCs.

The Simpson’s Diversity Index (S) was calculated as S = 1 − D, where D is the Simpson’s Index

D = Σ(n_i_ × (n_i_ − 1))/(N × (N − 1)),

where n_i_ is the number of strains belonging to CC_i_, and N is the total number of strains studied.

### 2.10. Statistics

Unless otherwise stated, Student’s *t* test was applied and the *p* value below 0.05 was considered statistically significant. The Wilcoxon signed-ranks nonparametric test was applied to evaluate the difference between two strain sets

## 3. Results

### 3.1. Phylogenetic Characterization of L. monocytogenes Strains Isolated from Raw Food Products in 2001–2005 and 2019–2020

All 45 strains were typed using the MLST database BIGSdb-*Lm* https://bigsdb.pasteur.fr/listeria/listeria.html (accessed on 10 September 2021). The 45 strains were prescribed to 14 sequence types (STs) belonging to distinct clonal complexes (CCs) (Table S1). Prescription of clonal complexes to phylogenetic lineages according to the MLST database BIGSdb-*Lm* https://bigsdb.pasteur.fr/listeria/listeria.html (accessed on 10 September 2021) showed that 14 and 31 strains belonged to the phylogenetic lineages I and II, respectively. There were no strains belonging to the phylogenetic lineages III and IV.

**Figure 2:**
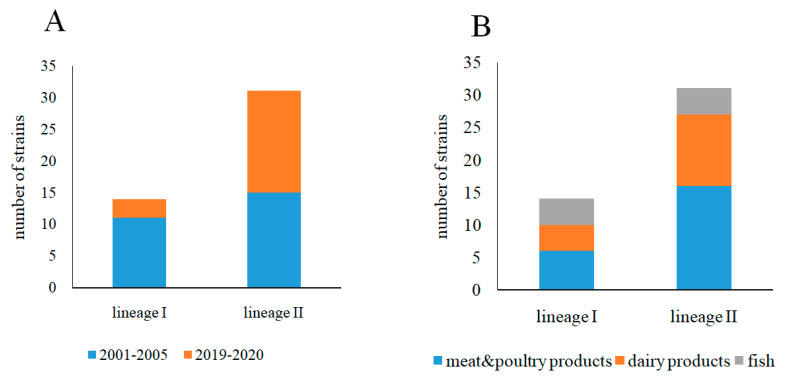
Representation of lineage I and lineage II strains. (**A**)—Among isolates obtained in 2001–2005/2019–2020 years; (**B**)—among isolates obtained from different food products.

The number of strains belonging to the lineage I and lineage II differed for the isolates obtained in 2001–2005 and 2019–2020. The majority, 11 of 14 lineage I strains were isolated in 2001–2005 (Figure 2A). The number of lineage II strains was approximately the same for the isolates obtained in 2001–2005 and 2019–2020 (15 and 16 strains in 2001–2005 and 2019–2020, respectively). Lineage I strains were distributed relatively evenly relative to the source of isolation (six, four, and four isolates were obtained from meat and poultry products, dairy products, and fish, respectively) (Figure 2B). The distribution relative to the source of isolation was different for the lineage II strains (16, 11, and 4 isolates from meat and poultry products, dairy products, and fish, respectively).

Serovar typing performed using the PCR approach described by Doumith et al. [29] revealed four serovars (Table S1). The prevailing serovar 1/2a was detected in 55% of isolates in all sources. Two and one *L. monocytogenes* strains of serovar 1/2b were isolated from fish in 2003 and from poultry products in 2019, respectively. Two strains from dairy products in 2005 and two strains isolated from meat and poultry products in 2019 belonged to serotype 1/2c. Nine *L. monocytogenes* strains were of the 4b serovar.

**Figure 3:**
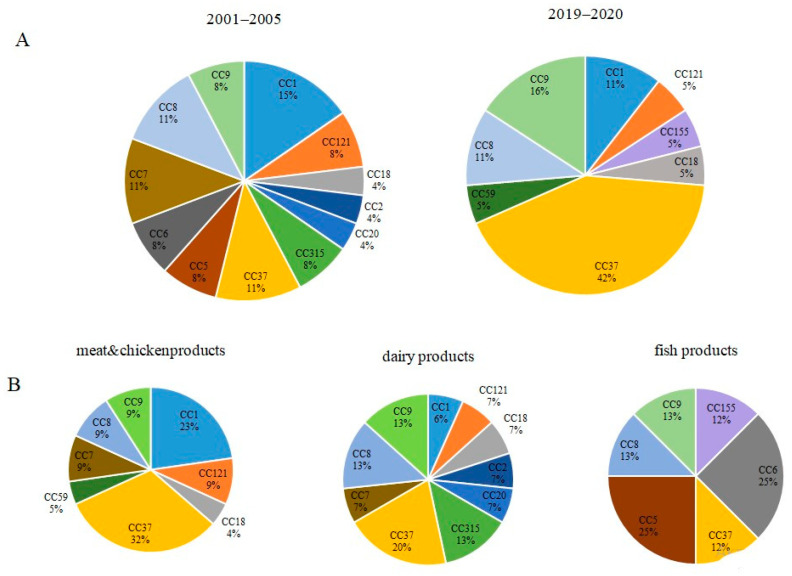
Phylogenetic diversity of studied groups. (**A**)—Diversity of strains isolated during 2001–2005 and 2019–2020. (**B**)—Diversity of strains isolated from different food products.

Totally, 12 and 8 CCs were recognized among strains isolated in 2001–2005 and 2019–2020, respectively (Table S2 and Figure 3A). Relative representation of CCs was almost uniform among strains isolated in 2001–2005. In total, 42% of strains isolated in 2019–2020 belonged to CC37 while other CCs represented from 5%to 16%. The Shannon’s Diversity Index, which is a measure of the degree of randomness in a set of data was 1.3 times more for strains isolated in 2001–2005 comparatively to strains isolated in 2019–2020 (Table S2). The Simpson’s Diversity Index that measures the probability that two randomly selected individuals belong to different groups, showed the higher value for strains isolated in 2001–2005 comparatively to strains isolated in 2019–2020 (0.94 vs. 0.78, respectively, Table S2). The close to 1 value of the Simpson’s Diversity Index suggested that the evenness of distribution of distinct CCs among strains isolated in 2001–2005 was statistically significant. The Wilcoxon signed-ranks test showed that the difference was significant at *p* < 0.10 (*p* = 0.075). These results supported the observation of greater diversity of strains isolated in 2001–2005 comparatively to strains isolated in 2019–2020.

The same tests were applied to strains isolated from different sources (Figure 3B and Table S2). A higher diversity of strains isolated from dairy products compared to meat and poultry products was demonstrated, but the difference was not statistically significant according to the Wilcoxon signed-ranks test because N (8) was not large enough for the distribution of the Wilcoxon W statistic to form a normal distribution. Therefore, it was not possible to calculate an accurate *p* value.

### 3.2. Occurrence of Worldwide Distributed Epidemic Clones among Studied Strains

**Figure 4:**
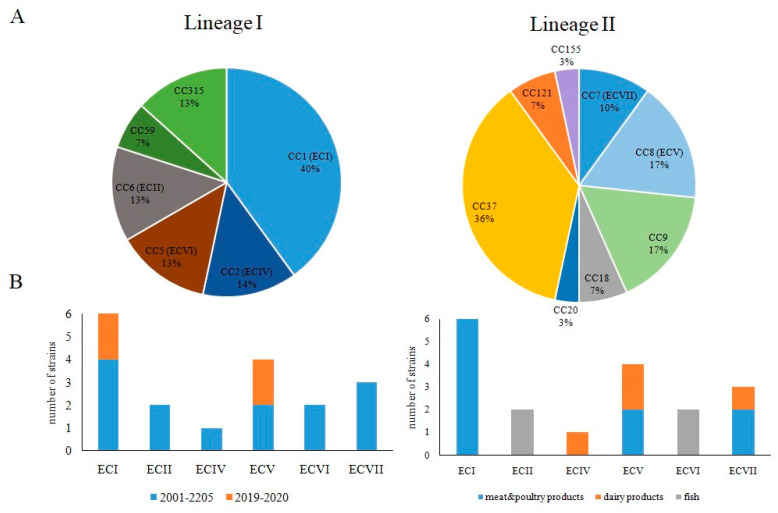
Frequency of clonal complexes and epidemic clones. (**A**)—Clonal complexes distribution among lineage I and lineage II strains; (**B**)—epidemic clones (ECs) among strains isolated from food products in 2001–2005/2019–2020.

The distribution of clonal complexes was uneven for lineage I and lineage II. In total, 40% lineage I strains belonged to the clonal complex CC1. The rest of the lineage I strains were approximately evenly distributed between the clonal complexes CC2, CC5, CC6, CC59, and CC315 (Figure 4A). The clonal complex CC37 prevailed among lineage II strains (36% of lineage II strains). Strains belonging to clonal complexes CC8, CC9, and CC7 (17%, 17%, and 10%, respectively) were next in the number of CC37 strains. The clonal complexes CC18, CC20, CC121, and CC155 were represented by one or two strains.

Six of seven described epidemic clones (ECs) that are responsible for multiple outbreaks of listeriosis worldwide were found among studied strains including lineage I: ECI, EC II, ECIV and ECVI, and lineage II: ECV and ECVII (Figure 4). ECI and ECV strains were isolated during both studied time periods, and ECII, ECIV, ECVI, ECVII strains were isolated in 2001–2005, but not in 2019–2020. All six ECI strains were isolated from meat and poultry products. ECIV, ECV, and ECVII strains were isolated from products of animal origin, while ECII and ECVI were isolated from fish (Figure 4B).

### 3.3. MvLST Typing Did Not Increase Resolution of the MLST Scheme but Showed Similarity of Invasion Factors InlA and InlB in Strains Belonging to Distinct CCs

**Figure 5:**
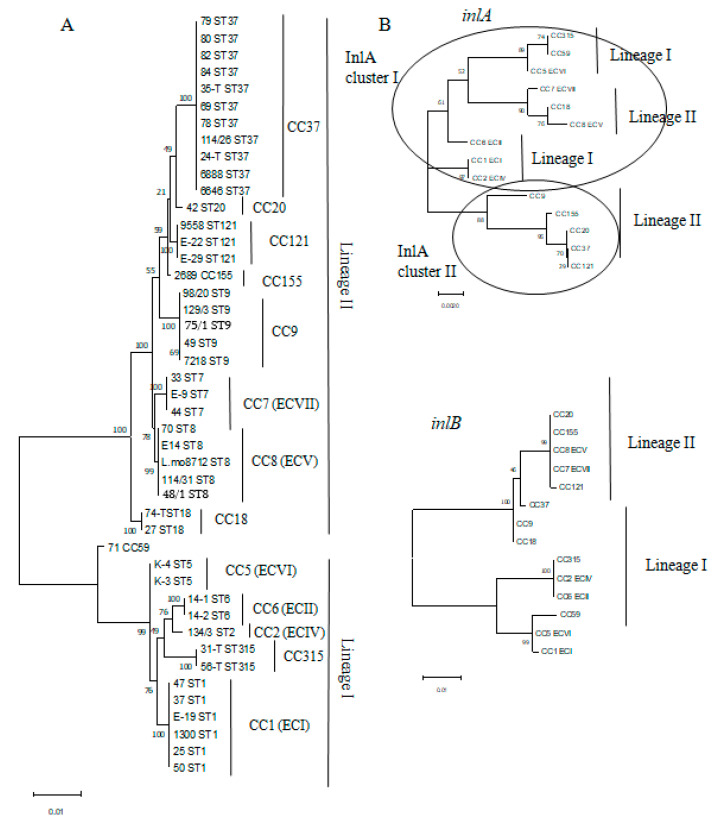
The maximal likelihood trees. (**A**)—The dendrogram based on the concatenated sequences of 11 markers included into the MvLST scheme; (**B**)—the dendrogram based on the sequences of the internal *inlA* and *inlB* gene fragments. The internal *inlA* fragments formed two clusters designated InlA cluster I and InlA cluster II.

Previously, we have demonstrated that internal loci of four virulence genes, that encode internaline proteins *inlA*, *inlB*, *inlC*, and *inlE* (the internalin profile, IP) can improve the MLST scheme discriminatory power [14,22]. We applied the combined MvLST (MLST + IP) scheme to characterize the studied strains. Concatenated sequences of all 11 genes formed two independent clusters corresponding to the phylogenetic lineages I and II on the maximum likelihood tree (Figure 5A). Strains were distributed along the tree in accordance with their belonging to MLST derived CCs. Strains belonging to the same CCs formed uniform isolated clusters demonstrating that application of the expanded scheme did not further improve resolution suggested by the MLST analysis.

To better characterize strains from the point of view of variability of major virulence factors InlA and InlB that determined the intestinal barrier crossing, we analyzed separately the sequences of *inlA* and *inlB* genes including into the MvLST profile (Figure 5B). The maximal likelihood tree demonstrated that while the *inlB* sequences formed two clusters corresponding to the phylogenetic lineages I and II in accordance with phylogenetic belonging of the strain they were found in, the *inlA* sequences included sequences characteristic for both lineage I and lineage II strains in a separate cluster which united strains belonging to epidemic clones ECV, ECVI, ECVII, and identical *inlA* gene fragments of ECI and ECII strains. This cluster was designated InlA cluster I. The *inlA* sequences from non-epidemic strains formed a separated cluster, designated InlA cluster II.

### 3.4. Invasion of L. monocytogenes into Human HEp-2 Cells

**Figure 6:**
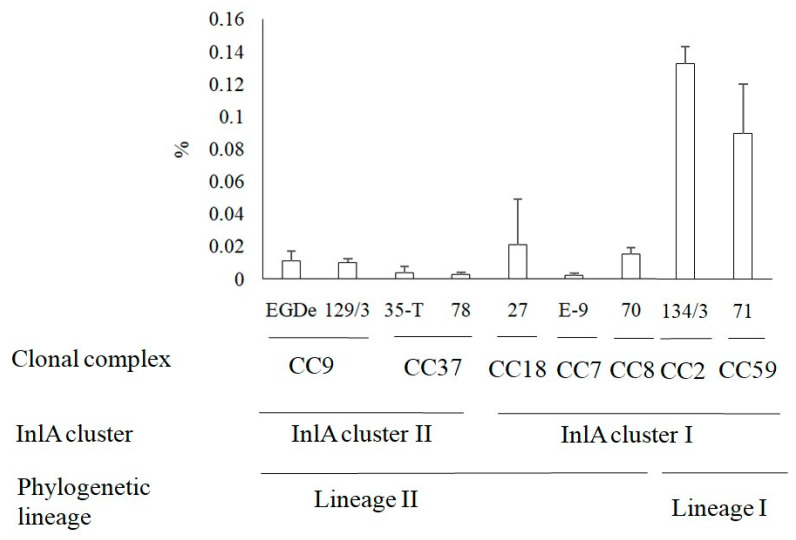
Invasion efficiency of *L. monocytogenes* into HEp-2 epithelial cells. The invasion coefficient values (ICs) measured as a percentage of invaded bacteria to the number of applied bacteria are shown. Totally, nine strains belonging to seven CCs were tested. ±SD value is based on three independent experiments. Statistical significance is described in the text.

The specific clustering of *inlA* sequences that was different from the phylogenetic dividing into lineages I and II and the known key role of InlA in intestinal barrier passage and human cell invasion led us to the idea to compare an invasion efficiency of different clonal complexes strains, phylogenetic lineages, and InlA clusters. In the present study, we studied the ability to penetrate into the cell of strains belonging to the most common clonal groups in our sample. The efficiency of invasion into human epithelial HEp-2 cells was studied in the gentamicin assay (see Section 2). The highest invasion efficiency was demonstrated by the CC2 strain Lmo134/3 (Figure 6; invasion coefficient IC = 0.13%). The lowest invasion efficiency was shown by the CC37 strain Lmo78 and the CC7 strain LmoE-9 (IC = 0.02% for both). There were no significant correlations between the year and/or source of isolation and the efficiency of the invasion. Invasion efficiency of the strains Lmo70, Lmo71, and Lmo78 isolated from poultry products in 2019 differed by more than 6 times (*p* < 0.05). The strains Lmo35-T and Lmo134/3 isolated from dairy products in 2005 were distinguished by 13 times (*p* < 0.01). Rather, genetic divergence could be responsible for different invasion efficiencies. Comparison of invasion coefficients of strains belonging to InlA cluster I and InlA cluster II did not reveal a significant difference. In contrast, the invasion efficiencies of lineage I strains were higher comparatively to lineage II strains (*p* < 0.05).

### 3.5. Antibiotics Resistance of L. monocytogenes

It is customary to use penicillin and ampicillin, or with the addition of gentamicin, in the treatment of human listeriosis. Exceptions are patients with an allergic reaction to penicillin who have been prescribed a combination of trimethoprim with a sulfamethoxazole [30,31]. The use of penicillin G/ampicillin concurrently with gentamicin is considered the best choice in the treatment of listeriosis [32]. Therefore, we tested resistance of the studied strains to these antibiotics (Table S1). All our *L. monocytogenes* strains turned out to be susceptible to gentamicin. The strain LmoSES 44 that was isolated from meat in 2002 in Moscow and belonged to CC7 demonstrated ampicillin resistance. The strain Lmo1300 obtained in Tula in 2005 from dairy products with the CC1 genetic characteristic showed resistance to penicillin G.

## 4. Discussion

*L. monocytogenes* isolated from food products in the central European part of Russia were studied. The important feature of the studied collection was prevalence of strains belonging to epidemic clones (ECs). In total, 20 of 45 strains belonged to six of seven described ECs. The predominance of epidemic clones among *L. monocytogenes* regardless of the source, has been demonstrated repeatedly [15,18,20,25]. These studies showed the temporal changes in the phylogenetic structure of dominate clones with ECI, ECIV, and ECVII clones persisting over decades and relatively recent expansion of ECII, ECIII, ECVI, and ECVI, which were frequently isolated after the year 1990 [15,18]. In our study, both persisting and relatively novel ECs were found among food isolates obtained in Russia after the year 2000 that is in line with the results obtained in other countries.

Temporal changes in the *L. monocytogenes* population structure was described both in the restricted frame of local processing plants, and in the global scale of multiyear strain isolation in a particular region or from a particular source [18,28,32,33]. The long lasting studies of populations within a particular processing plant show effects of sanitation procedures and/or changes in the production process [34,35,36]. Reasons underlying global changes in the population structure are less evident. A shift from *L. monocytogenes* serovar 4b to serovar 1/2a among strains causing human listeriosis occurred in Europe and Northern America after the year 2000 that was suggested to be due to changes in the consumer lifestyle and eating habits [17,28]. Increased consumption of RTE foods increases the occurrence of human listeriosis caused by strains persisting at the food processing plants. On the other hand, the increased world trade provides spreading of highly adaptive clones to the territories where they have been rare or not occurred before [25,27]. In Russia, the shift from phylogenetic lineage II to the phylogenetic lineage I was observed since the end of the XX century: while among strains associated with listeriosis in the second half of the 20th century almost 100% strains belonged to the lineage II, among human isolates of 2018–2020, the lineage II strains accounted for only 60% of human listeriosis [23,37].

Previous study of *L. monocytogenes* associated with human and animal disease has demonstrated the total domination of the lineage II strains with the prevalence of ECVII (CC7) in the territory of Russia and other countries of inner Eurasia between 1947 and 1990 [23]. Another study demonstrated that lineage II and ECVII (CC7) strains have persisted in the natural foci of infection situated in forests of the European part of Russia up to now [12]. However, in this study we demonstrated noticeable presentation of lineage I strains while only three ECVII strains were found among all isolates. This contradiction might be resolved if we speculate that abundance of clones in the food products after 2000 is determined by a combination of strains persisting in a particular region in the natural environments, strains incoming with the imported products, and strains incoming with the imported raw materials and further persisting in food chain environment while in the middle and the second third of the XX century a bacteria direct transmission from natural foci was the main route of *L. monocytogenes* delivery with the anthropogenic foci forming on farms. Obtained results support this suggestion. The Russian domestic food market was practically closed before the year 1990. Later the situation changed. The percentage of imported food materials increased up to 60–70% in the 2000s [27,38]. Particularly, we link the higher abundance of epidemic clones and higher representation of lineage I strains among 2001–2005 isolates by the full openness of the Russian food market in this period. However, in the years 2019–2020 the situation has changed again, and the share of imported food products decreased due to political and pandemic reasons. We suppose that the observed difference in diversity and EC presentation between strains isolated in 2000–2205 and 2019–2020,less representation of lineage I strains, and less variability of ECs among 2019–2020 isolates are correlated with the less open market. The increased proportion of the lineage II (serovar 1/2a) comparatively with lineage I (serovar 4b) strains among isolates obtained in 2019–2020 is in line with both the global tendency to the lineage II increased representation and increased presentation of strains circulating in the natural reservoirs situated in Russia under conditions of restricted food product import.

Comparison of the obtained results with isolates obtained from sporadic listeriosis cases in Moscow hospitals in 2018–2019 demonstrated a prevalence of lineage II strains (11 of 18 strains) among clinical isolates [37]. The ECVII (CC7) strains were the most frequent among human isolates obtained in Russia supporting the role of ECVII as a worldwide epidemic clone [22,38]. In this work, we analyzed sequence of internalin genes encoding the invasion factors InlA and InlB, which are key factors in intestinal barrier crossing and the disease development and found that the ECVII *inlA* gene fragment is combined into one cluster with sequences characteristic of lineage I ECs (see Figure 5B). Still, randomly selected ECVII strain invasion comparison and strains belonging to EC and non-EC clonal complexes did not support the idea that *inlA* gene similarities would provide high-efficiency *L. monocytogenes* invasion into human epithelial cells (see Figure 6). Other factors seem to affect invasion efficiency providing the higher invasion of lineage I strains comparatively to lineage II strains. Further, the sequenced *inlA* gene fragment encoded the part of the InlA LRR domain involved in interactions with the target mammalian receptor E-cadherin and the non-sequenced fragments might be more important for effective invasion [7].

Taken together, obtained results demonstrate high spreading of internationally recognized ECs among food isolates obtained in Russia after 2000. Further, our results show changes in representation of lineage I and lineage II strains among food isolates and raise the question about the potential reasons for these changes. Investigation of the pathways leading to the product contamination with strains of clonal complexes that are typical for a given region can be useful for stopping the penetration and fixation of highly virulent clones in territories new to them.

## Supplementary Materials

The following are available online at https://www.mdpi.com/article/10.3390/foods10112790/s1. Table S1: *Listeria monocytogenes* strains used in the study, Table S2: Diversity indexes of compared data sets.
