# Supplementary material for: Diversity of Listeria monocytogenes Strains Isolated from Food Products in the Central European Part of Russia in 2000–2005 and 2019–2020
Source: Foods. 2021 Nov 12;10(11):2790. doi: 10.3390/foods10112790 (PMC8617672; doi:10.3390/foods10112790)
Supplement: Supplementary file 1 [file foods-10-02790-s001.zip › Table S1.pdf]

Table S1. *Listeria monocytogenes* strains used in the study

N	Name of Strain	ID BIGSdb-*Lm* *	Year	Source	Location	Name of Serovar	ST	CC	*inlA*	*inlB*	*inlC*	*inlE*	IP**
1	L.mo14-1 33	78778	2001	fish	Saint Petersburg	4b	6	CC6	16	1	4	17	63
2	L.mo14-2 34	78779	2001	fish	Saint Petersburg	4b	6	CC6	16	1	4	17	63
3	L.moE14	78780	2002	fish	Moscow	1/2a	8	CC8	20	14	6	6	53
4	L.moSES 33	78781	2002	dairyproducts	Moscow	1/2a	7	CC7	4	14	6	8	15
5	L.moSES 37	78782	2002	meat	Moscow	4b	1	CC1	1	9	1	3	5
6	L.moSES 42	78783	2002	dairy products	Moscow	1/2a	20	CC20	9	14	6	6	13
7	L.moSES 44	78784	2002	meat	Moscow	1/2a	7	CC7	4	14	6	8	15
8	L.moSES 47	78785	2002	meat	Moscow	4b	1	CC1	1	9	1	3	5
9	L.moE-9	78786	2002	meat	Moscow	1/2a	7	CC7	4	14	6	8	15
10	L.moE-19	78787	2002	meat	Moscow	4b	1	CC1	1	9	1	3	5
11	L.moE-22	78788	2002	meat	Moscow	1/2a	121	CC121	9	17	7	2	48
12	L.moE-29	78789	2002	dairy products	Moscow	1/2a	121	CC121	9	17	7	2	48
13	L.moK-3	78790	2003	fish	Moscow	1/2b	5	CC5	3	8	4	1	38
14	L.moK-4	78791	2003	fish	Moscow	1/2b	5	CC5	3	8	4	1	38
15	L.mo74-T	78792	2005	dairy products	Tula region	1/2a	18	CC18	12	13	6	6	35
16	L.mo31-T	78793	2005	dairy products	Tula region	4b	194	CC315	2	11	4	1	64
17	L.mo8712	78794	2005	dairy products	Tula region	1/2a	8	CC8	20	14	6	6	53
18	L.mo98/20	78795	2005	dairy products	Tula region	1/2c	9	CC9	7	13	18	9	46
19	L.mo1300	78796	2005	dairy products	Tula region	4b	1	CC1	1	9	1	3	5
20	L.mo129/3	78797	2005	dairy products	Tula region	1/2c	9	CC9	7	13	18	9	46
21	L.mo114/31	78798	2005	dairy products	Tula region	1/2a	8	CC8	20	14	6	6	53
22	L.mo56-T	78799	2005	dairy products	Tula region	4b	194	CC315	2	11	4	1	64
23	L.mo114/26	78800	2005	dairy products	Tula region	1/2a	37	CC37	9	11	7	8	28
24	L.mo24-T	78801	2005	dairy products	Tula region	1/2a	37	CC37	9	11	7	8	28
25	L.mo35-T	78802	2005	dairy products	Tula region	1/2a	37	CC37	9	11	7	8	28
26	L.mo134/3	78803	2005	dairy products	Tula region	4b	2	CC2	1	1	1	1	1
27	L.mo25	79358	2019	chicken	Bryansk region	4b	1	CC1	1	9	1	3	5
28	L.mo27	78804	2019	meat	Moscow region	1/2a	18	CC18	12	13	6	6	35
29	L.mo48/1	data processing	2019	meat	Moscow region	1/2a	8	CC8	20	14	6	6	53

30	L.mo49	79359	2019	chicken	Moscow region	1/2c	9	CC9	7	13	18	9	46
31	L.mo50	79360	2019	chicken	Moscow region	4b	1	CC1	1	9	1	3	5
32	L.mo69	79361	2019	chicken	Moscow region	1/2a	37	CC37	9	11	7	8	28
33	L.mo70	78808	2019	chicken	Moscow region	1/2a	8	CC8	12	14	6	6	34
34	L.mo71	78809	2019	chicken	Moscow region	1/2b	59	CC59	2	8	14	3	65
35	L.mo75/1	data processing	2019	meat	Moscow region	1/2c	9	CC9	7	13	18	9	46
36	L.mo78	79363	2019	chicken	Tver region	1/2a	37	CC37	9	11	7	8	28
37	L.mo79	79364	2019	chicken	Tver region	1/2a	37	CC37	9	11	7	8	28
38	L.mo80	79365	2019	meat	Moscow region	1/2a	37	CC37	9	11	7	8	28
39	L.mo82	79366	2019	meat	Moscow region	1/2a	37	CC37	9	11	7	8	28
40	L.mo84	79367	2019	meat	Moscow region	1/2a	37	CC37	9	11	7	8	28
41	GIMC2035:Lmc7218	45731	2019	fish	Moscowregion	n.d.***	9	CC9	7	13	18	9	46
42	GIMC2024:Lmc2689	75930	2019	fish	Moscow	n.d.	155	CC155	10	14	19	2	50
43	GIMC2054:Lmc6888	49372	2020	chicken	Belgorod	n.d.	37	CC37	9	11	7	8	28
44	GIMC2053:Lmc6646	49371	2020	fish	Moscowregion	n.d.	37	CC37	9	11	7	8	28
45	GIMC2059:Lmc9558	75930	2020	chicken	Moscow	n.d.	121	CC121	9	17	7	2	48

* BIGSdb-*Lm* provides access to genotypic data for *Listeria* isolates based on Multilocus Sequence Typing (MLST)

**IP is the internalin profile formed from genes encoding proteins of the internalin family *inlA*, *inlB*, *inlC*, and *inlE*

*** n.d. - no defined
