# Supplementary material for: Diversity of Listeria monocytogenes Strains Isolated from Food Products in the Central European Part of Russia in 2000–2005 and 2019–2020
Source: Foods. 2021 Nov 12;10(11):2790. doi: 10.3390/foods10112790 (PMC8617672; doi:10.3390/foods10112790)
Supplement: Supplementary file 1 [file foods-10-02790-s001.zip › Table S2.pdf]

Table S2. Diversity indexes of compared data sets

Strain Set	N Strains	N CCs	Shannon's Diversity Index	Simpson's Index (D)	Simpson's Diversity Index
2001–2005	26	12	3.5	0.06	0.94
2019–2020	19	8	2.7	0.22	0.78
